# Isolated enophthalmos: an uncommon gateway to orbital tumors in pediatrics: 9 month-old female presenting with isolated enophthalmos as the unique sign of a metastatic orbital tumor: a case report

**DOI:** 10.1186/1471-2431-14-237

**Published:** 2014-09-23

**Authors:** Sara Touhami, Emmanuel Bui-Quoc

**Affiliations:** Ophthalmology Department, La Pitié Salpétrière Hospital, 47-83 Boulevard de l’Hôpital, 75013 Paris, France; Pediatric Ophthalmology Department, Robert Debré Hospital, 48 Boulevard Serurier, 75019 Paris, France

**Keywords:** Enophthalmos, Exophthalmos, Proptosis, Infantile orbital tumors, Neuroblastoma, Pediatric tumors

## Abstract

**Background:**

If extra-axial proptosis is by far the most common symptom of infantile malignant orbital tumors, enophthalmos is a rare and undocumented sign. We report the first case of a pediatric metastatic orbital tumor revealed by enophthalmos alone.

**Case presentation:**

A 9-month-old girl was diagnosed with isolated right-sided enophthalmos. An orbital tumor was suspected and computed tomography undertaken showing osteolysis and periosteal reaction of orbital walls, malar bones and zygomatic arches. A Thoracic- abdominal CT scan confirmed a stage-4 neuroblastoma.

**Conclusion:**

Enophthalmos can be the sole symptom of an orbital tumor and should lead to immediate imaging assessment. This association is not well known in pediatrics but is relevant to insure the best prognosis.

## Background

Exophthalmos is the most common symptom of orbital tumors in adults and children given the narrowness of their bony structures. However, more scarce signs need to be recognized. Among these, enophthalmos has already been described in adults [1] but has never been reported as a sole indicator of orbital neoplasms in pediatrics. We report the first case of a pediatric metastatic neuroblastoma revealed by enophthalmos alone and stress the importance of this sign as a revealing symptom of orbital tumors in children.

## Case presentation

**Figure 1:**
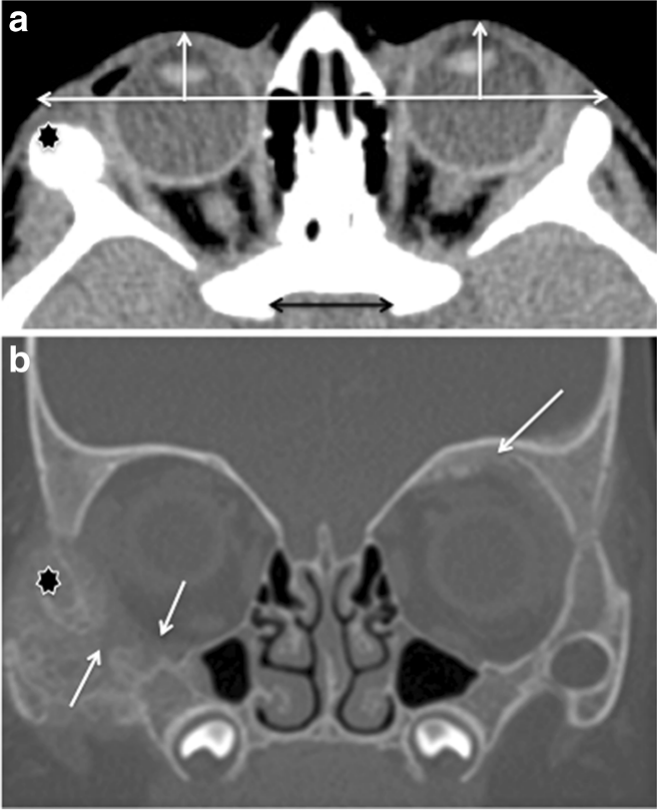
**CT Scan showing enophthalmos of the right eye and the causal neoplastic process. a**. CT cross-section showing a backwards displacement of the right eye comparatively to the left eye (Vertical white arrows). The baseline white bar does not sit on the lateral bony wall edge on the right side because of the osteolytic neoplastic process that compromises the integrity of the right zygomatic arch making it seem smaller but thicker and more heterogeneous (Black star, also in Figure 1b). The quality of the baseline bar’s horizontality was ensured by comparing it with the horizontal black double arrow that serves as point of reference. **b**. CT scan showing irregularly shaped osteolysis of both orbital walls, malar bones and zygomatic arches (Black star on the right side) with periosteal reaction. The orbital cavity seems to be increased on the right side as compared with the left side. The osteolytic process induces cracks and fractures on the inferior wall of the right orbit (White arrows), and the osteocondensation with bone neoformation at the superior wall of the left orbit (white arrow) associated with soft tissue inflammation, denotes less space available for the right globe as compared with the left globe, which could explain the enophthalmos of the right eye.

**Figure 2:**
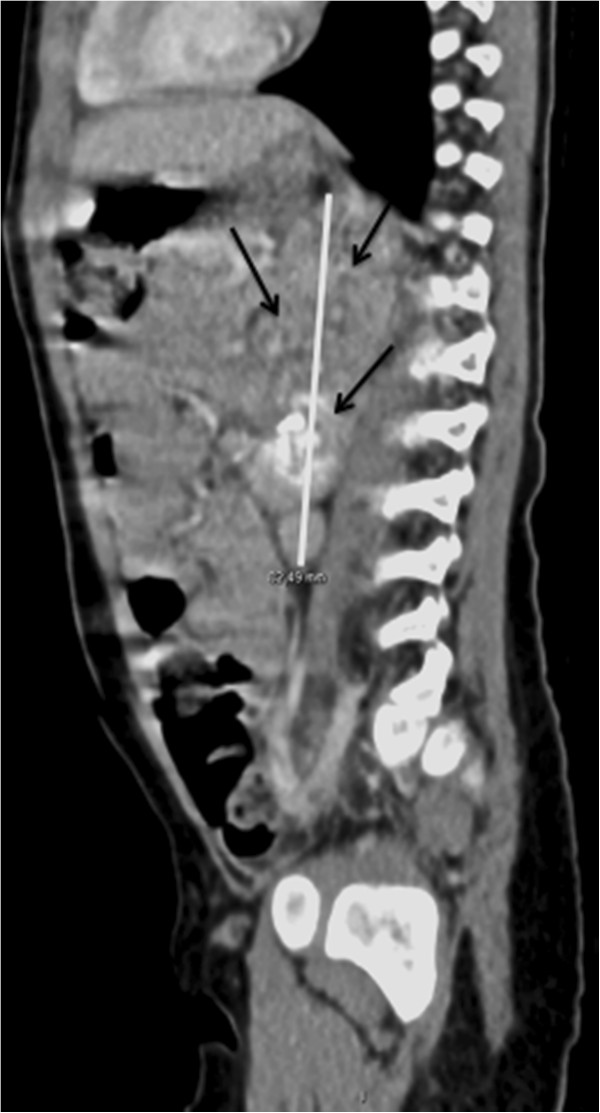
Thoracic- abdominal CT scan revealing a 83 mm*43 mm*42 mm retroperitoneal calcified heterogeneous mass (White Thick Bar) located on the left adrenal gland and perirenal region evocative of neuroblastoma (Black Arrows).

A 9-month-old Caucasian female without any birth or past medical history was diagnosed with isolated enophthalmos of the right eye. The mother had noticed a backwards displacement of the right eye 3 months prior to presentation and consulted various specialists who stated a constitutional feature. General examination was normal with no deterioration of health status, neurological, abdominal or skeletal bone integrity. Ophthalmological examination revealed nothing but a mild right enophthalmos. There was no facial disfigurement such as flattening. Pupil size and reactivity to light, direct and consensual accommodation, fixation and following, binocular function, eye-hand coordination, reaction to patching, slit lamp and fundus examination were all normal. Before this isolated enophthalmos, blood and urine samples were collected and orbital computed tomography (CT) undertaken. Routine biology labs, in particular creatinin levels, were normal. An orbital CT scan confirmed the enophthalmos (Figure 1a) and showed irregularly shaped osteolysis with periosteal reaction of orbital walls, malar bones and zygomatic arches, predominantly on the right side (Figure 1b), arousing suspicion of a tumor. A thoracic- abdominal CT scan showed an 83 mm*43 mm*42 mm retroperitoneal heterogeneous mass located on the left adrenal gland (Figure 2) embracing vascular elements and associated with intra-abdominal, left supraclavicular swollen lymph nodes and vertebral condensations, which was evocative of a stage-4 neuroblastoma. The patient was immediately referred to oncology for further investigation and treatment. The stage-4 neuroblastoma was confirmed and the patient treated with chemotherapy and surgical evacuation of residual masses. She achieved complete remission with no recurrence at 10-month follow-up.

## Discussion

Exophthalmos is the most commonly known symptom of orbital tumors in adults and children [2] and is, usually easy to recognize, and always brings to mind the possibility of a neoplasm. Conversely, if a few cases have been described in adults [1, 3–5], there is only very little knowledge of the association between enophthlamos and orbital tumors in children [2] ; because of the scarcity of both entities and the difficulty in clinically objectivizing this symptom. In fact, enophthalmos is a posterior displacement of the eyeball within the orbit but its diagnosis is tricky because there is no agreement on a clear definition, especially in children. For example, Yip set a 14 mm limit [6] while diagnosis is not made formal until after orbital CT scan measures an oculo-orbital index (OOI) < 30 % (OOI = Prebicanthal eyeball length/overall eyeball length x100). Nonetheless, a large majority of specialists agree on the importance of clinical and exophthalmometric judgment over imaging assessment.

When this symptom is identified, the next step is to rule out differential diagnoses: contralateral proptosis, ipsilateral ptosis (including Horner syndrome), microphthalmia and phthisis bulbi.

Once those are excluded, the cause needs to be sought. MacFaul stated in his “System of ophthalmology” that unlike exophthalmos^,^ enophthalmos could not lead to a fatal outcome [7]; because his classification failed to mention orbital tumors as a possible etiology. He stated that orbit topography is such that infiltrative processes are more likely to push outwards and induce proptosis [3]. Most of the time, this statement is true, however in rare cases; neoplastic cells can infiltrate extraocular muscles, alter orbital fat structure and destroy bony architecture leading to a backwards eyeball traction [4].

Based on this, three possible mechanisms have been suggested to explain the occurrence of enophthalmos [4]. First, structural modifications: post traumatic bone fractures, congenital bony defects; silent sinus syndrome and tumors can crack the orbit wall and modify the eyeball position [4, 5]. Second: eyeball backwards retraction consecutive to muscle and/or fat tissue infiltration [4, 5]. Fitting into this category are adult cases of enophthalmos, which can reveal various types of orbital neoplasms. Affected patients are usually females with breast cancer [1, 5]. When the musculature is invaded, areas of fibrosis are created causing posterior traction of the eyeball. In such cases, enophthalmos is usually not isolated and is associated with a palpable mass, impaired eye motility, diplopia, orbital pain, drooping of the upper eyelid etc.. [1, 3–5]. Third: fat atrophy (such as in senile fat atrophy and orbital varices) might induce an eyeball displacement by shrinking the orbital content [4, 5].

In the present case, the three mechanisms could have caused a posterior displacement of the eyeball. By cracking the orbital wall (Figure 1b), the neuroblastoma modified the eyeball position dragging it downwards and backwards. Additionally, the orbital cavity seemed to be increased on the right side as compared with the left side. In Figure 1b, the osteolytic process induced fractures of the right orbit’s inferior wall and osteocondensation with bone neoformation at the superior wall of the left orbit associated with soft tissue inflammation, which denotes less space available for the right globe as compared with the left globe, possibly explaining the enophthalmos of the right eye. Additionally, muscle and fat infiltration by the neuroblastoma could have induced local areas of retractile fibrosis pushing the globe backwards, as shown in Figure 1b where the right globe seems to be smaller than the left globe whereas the cross-section is perfectly vertical, denoting a backwards retraction of the right eye. Third, (though this is probably less likely), the growth of the neuroblastoma could have induced a shrinking of the orbit’s other components including fat. As seen in Figure 1a, the orbital content is less prominent in the right side as compared to the left side, causing a backwards displacement of the eye.

## Conclusion

Enophthalmos can be the only symptom of a neoplastic process and should lead to immediate imaging assessment to rule out an orbital tumor and avoid the disastrous outcome of a delayed diagnosis.

## Consent

All examinations and investigations in this case report followed the tenets of the Declaration of Helsinki. The ethics committee of the French Society of Ophthalmology also approved them. Informed consent was obtained from the patient’s legal guardians for publication of this case report and accompanying images.
